# Prenylation Inhibition-Induced Cell Death in Melanoma: Reduced Sensitivity in BRAF Mutant/PTEN Wild-Type Melanoma Cells

**DOI:** 10.1371/journal.pone.0117021

**Published:** 2015-02-03

**Authors:** Tamás Garay, István Kenessey, Eszter Molnár, Éva Juhász, Andrea Réti, Viktória László, Anita Rózsás, Judit Dobos, Balázs Döme, Walter Berger, Walter Klepetko, József Tóvári, József Tímár, Balázs Hegedűs

**Affiliations:** 1 2nd Department of Pathology, Semmelweis University, Budapest, Hungary; 2 National Koranyi Institute of TB and Pulmonology, Budapest, Hungary; 3 Department of Biological Physics, Eötvös University, Budapest, Hungary; 4 Department of Thoracic Surgery, Medical University of Vienna, Vienna, Austria; 5 Department of Experimental Pharmacology, National Institute of Oncology, Budapest, Hungary; 6 Department of Thoracic Surgery, Semmelweis University-National Institute of Oncology, Budapest, Hungary; 7 Institute of Cancer Research and Comprehensive Cancer Center, Medical University of Vienna, Vienna, Austria; 8 MTA-SE Molecular Oncology Research Group, Hungarian Academy of Sciences, Budapest, Hungary; The Moffitt Cancer Center & Research Institute, UNITED STATES

## Abstract

While targeted therapy brought a new era in the treatment of BRAF mutant melanoma, therapeutic options for non-BRAF mutant cases are still limited. In order to explore the antitumor activity of prenylation inhibition we investigated the response to zoledronic acid treatment in thirteen human melanoma cell lines with known BRAF, NRAS and PTEN mutational status. Effect of zoledronic acid on proliferation, clonogenic potential, apoptosis and migration of melanoma cells as well as the activation of downstream elements of the RAS/RAF pathway were investigated *in vitro* with SRB, TUNEL and PARP cleavage assays and videomicroscopy and immunoblot measurements, respectively. Subcutaneous and spleen-to-liver colonization xenograft mouse models were used to evaluate the influence of zoledronic acid treatment on primary and disseminated tumor growth of melanoma cells *in vivo*. Zoledronic acid more efficiently decreased short-term *in vitro* viability in NRAS mutant cells when compared to BRAF mutant and BRAF/NRAS wild-type cells. In line with this finding, following treatment decreased activation of ribosomal protein S6 was found in NRAS mutant cells. Zoledronic acid demonstrated no significant synergism in cell viability inhibition or apoptosis induction with cisplatin or DTIC treatment *in vitro*. Importantly, zoledronic acid could inhibit clonogenic growth in the majority of melanoma cell lines except in the three BRAF mutant but PTEN wild-type melanoma lines. A similar pattern was observed in apoptosis induction experiments. *In vivo* zoledronic acid did not inhibit the subcutaneous growth or spleen-to-liver colonization of melanoma cells. Altogether our data demonstrates that prenylation inhibition may be a novel therapeutic approach in NRAS mutant melanoma. Nevertheless, we also demonstrated that therapeutic sensitivity might be influenced by the PTEN status of BRAF mutant melanoma cells. However, further investigations are needed to identify drugs that have appropriate pharmacological properties to efficiently target prenylation in melanoma cells.

## Introduction

Melanoma is characterized by high mortality among solid tumors due to the very high metastatic potential of melanoma cells and their resistance to therapy especially at late stage diseases [1, 2]. The three-year survival among patients with visceral metastases is less than 20% [3, 4].

Importantly, the majority of melanoma cases demonstrate oncogenic activation of the KIT—NRAS—BRAF—MEK—ERK central axis [5] that is a major regulator of cell differentiation and proliferation [6, 7]. The importance of this pathway is highlighted by the finding that BRAF and NRAS mutation are the two most important oncogenic mutations in melanoma and both of these mutations result in the constitutive activation of the RAS-RAF-MEK-ERK signaling cascade. BRAF mutation is detected in about 40 to 70% of the cases while NRAS mutation is present in 10 to 30% of melanomas [8–15]. In addition, RAS activates also the protein kinase B/Akt pathway where PTEN, a tumor-suppressor, acts as an endogenous inhibitor by catalyzing the PIP3 to PIP2 transformation thus counteracting PI3K [16]. PTEN-null mutations are present in 20% of melanoma cases [17, 18] furthermore PTEN null mutation is often concurrent with BRAF mutation in melanoma [19].

Accordingly, inhibitors of the RAS-RAF-MEK-ERK pathway carry great promises for anticancer treatment. However, due to the mechanism of Ras activation and signal transmission the direct targeting of the Ras protein is rather difficult [20]. Ras protein needs to be processed in the endoplasmic reticulum and transported to the cell membrane to exert its function. Thus, the posttranslational modification and the anchorage to the cell membrane of Ras are among the most intensely targeted steps in Ras-related tumor treatments [21].

For instance, S-farnesylthiosalicylic acid (FTS, Salirasib) competes with Ras for Ras-anchorage sites at the cell membrane and reduces Ras-dependent tumor growth [22]. However, the mechanism and the selectivity against activated Ras is still under investigation [23, 24]. One approach is the inhibition of farnesyltransferases that results in the inhibition of the thioether linked addition of an isoprenyl group to the CAAX-box cystein of Ras. These inhibitors showed great promise in preclinical models but failed to succeed in monotherapy clinical trials [25, 26]. One reason for the failure of this approach is that in human cancer cells treated with farnesiltransferase-inhibitors (FTIs), K-Ras and possibly N-Ras (but not H-Ras) become geranylgeranylated [27–29]. As a consequence, the blockade of Ras activation requires the inhibition of both farnesyltransferase and geranylgeranylase [30].

Bisphosphonates, a class of synthetic analogues of the endogenous pyrophosphate, inhibit the posttranslational modification of Ras proteins by blocking the intracellular key enzyme of the mevalonate pathway, farnesyl diphosphate syntase. This enzyme is responsible for the production of cholesterol and isoprenoid lipids such as farnesyl diphosphate and geranylgeranyl diphosphate [31, 32]. These isoprenoids are necessary for the post-translational lipid modification (prenylation) of Ras proteins including both farnesylation and geranylgeranylation. One of the clinically applied amino-bisphosphonates is zoledronic acid that is currently established in the treatment for bone lesions and metastases of a number of cancers.

An increasing number of both *in vitro* and *in vivo* studies suggest that bisphosphonates, especially nitrogen containing bisphosphonates such as pamidronate or zoledronate, may also have a specific antitumoral activity. Bisphosphonates have shown potential to inhibit proliferation, induce cell cycle changes and/or induce apoptosis in several studies on myeloma cells both *in vitro* [33–37] and *in vivo* [38]. Similar effects of bisphosphonate treatment were reported for osteosarcoma, prostate or breast carcinoma cells [34, 39–43]. In preclinical studies cancer types without preferential spreading to bone were also treated effectively with bisphosphonates. Pancreatic cancer cells [44] and even neural crest derived neuroblastoma cells [45] have shown sensitivity to zoledronic acid treatment. Very recently, zoledronic acid was proven to enhance the effect of EGFR-inhibitors and even to overcome resistance acquired through the gatekeeper mutation T790M in non-small cell lung cancer, breast cancer and colorectal cancer [46, 47].

In melanoma the aminobisphosphonate pamidronate was able to inhibit proliferation and induce apoptosis [48]. Furthermore the treatment of melanoma cells with nitrogen-containing bisphosphonates (pamidronate and zoledronate), but not the treatment with nonaminobisphosphonates (clodronate) resulted in decreased cell proliferation *in vitro* [49]. Nevertheless, the effect of zoledronic acid on melanoma cells *in vivo* and the dependence of biological response on the BRAF or NRAS oncogenic mutation status have not yet been studied.

Accordingly, we determined the impact of BRAF, PTEN and NRAS mutation on the effect of zoledronic acid treatment on cell viability, clonogenic growth, apoptosis induction, cell migration and activation of the downstream signaling pathway in melanoma cells *in vitro* and on primary tumor growth and spleen-to-liver colonization in mice models of human melanoma cells *in vivo*.

## Methods

### Cell lines and culture conditions

Thirteen human melanoma cell lines were used in the experiments. HT168-M1 melanoma line was a highly metastatic derivative from the A2058 cell line isolated in liver colonization model of mice [50]. The BRAF mutant/PTEN-null HT199 melanoma cell line was developed at the Semmelweis University, Budapest Hungary [51]. The M24met melanoma line (NRAS mutant), established from an invaded lymph node of a nude mouse [52], was kindly provided by B. M. Mueller (Scripps Research Institute, La Jolla, CA). VM-15 (NRAS mutant) and VM-47 (wild-type) cell lines were established at Institute of Cancer Research, Vienna, Austria from lymph node and brain metastasis, respectively [53, 54]. The melanoma cell lines A375, A2058, LOX (BRAF mutant) and MEWO (wild-type) are available from ATCC. WM3060, WM3670 (NRAS/BRAF mutant), WM35 and WM239 cell lines were obtained from the Wistar Institute. The mutational status of cell lines is presented in Table 1. Cell cultures were maintained in DMEM (Lonza, Switzerland; with 4500mg/dm^3^ glucose, piruvate and L-glutamine) supplemented with 10% fetal calf serum (Lonza) and 1% penicillin-streptomycin-amphotericyn (Lonza) in tissue culture flasks at 37C in a humidified 5% CO_2_ atmosphere.

**Table 1:** Mutational status of all 13 human melanoma cell lines.

	BRAF	NRAS	PTEN	REFERENCE
MEWO	wt	wt	+	[74](NF1 mutant)
VM47	wt	wt	+ (weak)	own sequence
WM3060	wt	61K	+	[75]
VM15	wt	61K	+ (weak)	own sequence
M24met	wt	61R	+	own sequence
WM3670	G469E	12D	+	[75]
WM35	V600E	wt	+	[65, 76]
LOX	V600E	wt	+	[77]
A375	V600E	wt	+	[77]
HT168-M1	V600E	wt	-	own sequence
A2058	V600E	wt	-	[77]
WM239	V600E	wt	-	[65, 76]
HT199	V600E	wt	-	own sequence

### Mutational analysis

PCR reaction to multiply BRAF and NRAS genes were performed with from the cell lines isolated DNA in cell lines with no previously published mutational status. In a consecutive step samples were purified with Applied Biosystems BigDye XTerminator Purification Kit and mutations were verified through sequencing on Abi 3130 Genetic Analyzer System with BigDye Terminator v1.1 Kit. Loss of PTEN was shown using immunoblot analysis (S1 Fig.).

### Proliferation (SRB) assay

To analyze short time effect of zoledronic acid (ZA) on cell proliferation Sulforhodamine B (SRB) assay was performed based on the measurement of cellular protein content. Briefly, melanoma cells were plated in the inner 60 wells of a 96-well plate. After 24hs in control medium and 72hs treatment with zoledronic acid, cell monolayers were fixed with 10% trichloroacetic acid and stained for 15 min with SRB. Cells were repeatedly washed with 1% (vol/vol) acetic acid to remove excess dye. The protein-bound dye was dissolved in 10 mM Tris and OD determined at 570 nm using a microplate reader (EL800, BioTec Instruments, Winooski, VT). Data shown as average of six independent experiments and effect of treatment is expressed relative to control.

### Clonogenic assay

Long time antiproliferative effect of ZA treatment was assessed by performing clonogenic assay. Briefly, 1000 cells (for LOX, WM3060, WM3670, VM-47 cells 10000 cells) were seeded in 6 well plates and treated 5μM ZA for 10 days. Fresh medium and ZA were supplied on each 3^rd^ day. On the 10^th^ day cells were fixed with 10% trichloroacetic acid and stained and measured as in the SRB assay.

### Apoptosis detection by TUNEL-staining

For apoptosis detection cells were set on 24 well plates and treated with either zoledronic acid and/or a cytotoxic agent (DTIC or cisplatin). After 48hs of treatment cells were fixed with 4% buffered PFA and labeling of terminal deoxynucleotidyl transferase—mediated dUTP nick end labeling (TUNEL) was performed according to the manufacturer’s instructions (Roche Diagnostics, Basel, Switzerland). Quantification was done by direct counting of the TUNEL-positive cells on at least five 20× microscopic fields.

### Time-lapse videomicroscopy

Videomicroscopy measurements were performed and analyzed as described previously [55–57]. Briefly, melanoma cells were plated in the inner 8 wells of 24-well plates (Corning Incorporated, Corning, NY) in DMEM medium supplemented with 10% FCS. The medium was changed to CO_2_-independent medium (Gibco-BRL Life Technologies, Carlsbad, CA) with 10% FCS and 4mM glutamine after the overnight cell attachment. In order to reduce evaporation from the inner wells, the outer wells were filled with medium. Cells were kept in a custom designed incubator built around an inverted phase-contrast microscope (World Precision Instruments, Sarasota, FL) at 37°C and room ambient atmosphere. Images of 3 neighboring microscopic fields were taken in every 5 min for 1 day before and 2 days after the treatment with zoledronic acid, dacarbazine or both. For migration data the captured phase contrast microscope pictures were analyzed individually with a cell-tracking program enabling manual marking of individual cells and recording their position parameters into data files. The parameter migrated distance is calculated by averaging for each cell the displacement for the first 24 hour interval after treatment, in two independent experiments and 3 microscopic fields.

### Immunoblot analysis

Immunoblot analysis was performed to examine the expression of PTEN, the induction of apoptosis by PARP cleavage and the phosphorylation of Erk1/2 and S6 proteins, two downstream targets of the RAS pathway. Cells were set in six-well plates and maintained as mentioned above. After two days of incubation time cells were treated in two independent replicates for 12h with zoledronic acid, dacarbazin or both for the measurement of the activation of downstream effectors ERK1/2 and S6. 48hs ZA treatment was applied for the assessment of apoptosis and untreated cells were used for the evaluation of PTEN expression on the protein level. Cells were lysed with RIPA Buffer (Thermo Scientific, Waltham, MA) supplemented with 1% Halt Protease Inhibitor Single-Use Cocktail (Thermo Scientific). Total protein concentrations were determined with Pierce BCA Protein Assay kit (Thermo Scientific), denatured and equal amounts of protein were size-fractionated by SDS-PAGE (12%) and transferred to nitrocellulose membrane (Whatman, Maidstone, UK) The membranes were incubated with anti PTEN (Cell Signaling; # 9552), anti cleaved-PARP (Cell Signaling; # 9541) and anti p-Erk/Erk, p-S6/S6, p-Akt and as loading control anti β-tubulin (Cell Signaling; #9101, #9102, #2215, #2217, #4058 and # 2128 respectively), overnight at 4°C in a dilution of 1:2000. Secondary, HRP labeled anti-rabbit antibody was applied 1:2000 for 0,5h at room temperature. Visualization was achieved with Amersham ECL Advance Western Blotting Detection kit (GE HealthCare, Little Chalfont, UK). Activation of signaling was quantified as the ratio of phosphorylated and total protein densitometry measurements using ImageJ software.

### Subcutaneous xenografts of human melanoma cells

All animal-model protocols were carried out in accordance with the Guidelines for Animal Experiments and were approved for the Department of Experimental Pharmacology in the National Institute of Oncology, Budapest, Hungary (permission number: 22.1/722/3/2010).

HT168-M1, M24met and MEWO human melanoma cells (10^6^ HT168-M1 and MEWO, 10^5^ M24met) were subcutaneously injected in male SCID mice, at a weight of 30–33g and 10 animals per group. Since the HT168-M1 xenografts tend to outgrew rapidly mice transplanted with HT168-M1 were treated and sacrificed earlier. After randomization, animals were treated intraperitoneally weekly for three weeks. The treatment with zoledronic acid started when first tumors were measurable, consequently at day 10 for mice injected in with HT168-M1 or after two weeks for animal treated with M24met or MEWO. Controls received 100 μl of 0.9% NaCl. The subcutaneous tumors were measured with a caliper and tumor-volumes were calculated with the formula for the volume of a prolate ellipsoid (length ×width^2^ π/6) and expressed in cm^3^. After the last measurement of subcutaneous tumors animals were sacrificed by cervical dislocation.

### Spleen to liver colonization assay

Melanoma cells (5×10^2^ HT168-M1, 10^5^ M24met or 10^6^ MEWO) were injected into the spleen of male SCID mice under Nembutal anesthesia, 10 animals per group. Zoledronic acid (500 μg/kg) or saline as control was administered intraperitoneally from the 7^th^ day for HT168-M1 and from the 10^th^ day for M24met and MEWO cells post injection day and continued for 3 weeks. After 3 weeks of treatment animals were sacrificed by cervical dislocation, spleen and liver were removed and total weight of organs assessed.

### Statistics

To determine statistical differences between groups, ANOVA was performed for datasets with normal distribution and Dunnett’s post hoc test and Tukey’s post hoc test were computed if comparison to control or to each treated group, respectively, was questioned. Otherwise, non-parametric Kruskal-Wallis and post hoc Dunn’s multiple comparison test was used. Unpaired T test was calculated for the statistical evaluation of the treatment, if only one treatment was analyzed. Statistical significance was established at p < 0.05. All statistical analyses were computed in GraphPad Prism 5 (GraphPad Software Inc, USA, San Diego, CA).

## Results

### Mutational status dependent inhibition of *in vitro* growth by zoledronic acid (ZA)

**Fig 1:**
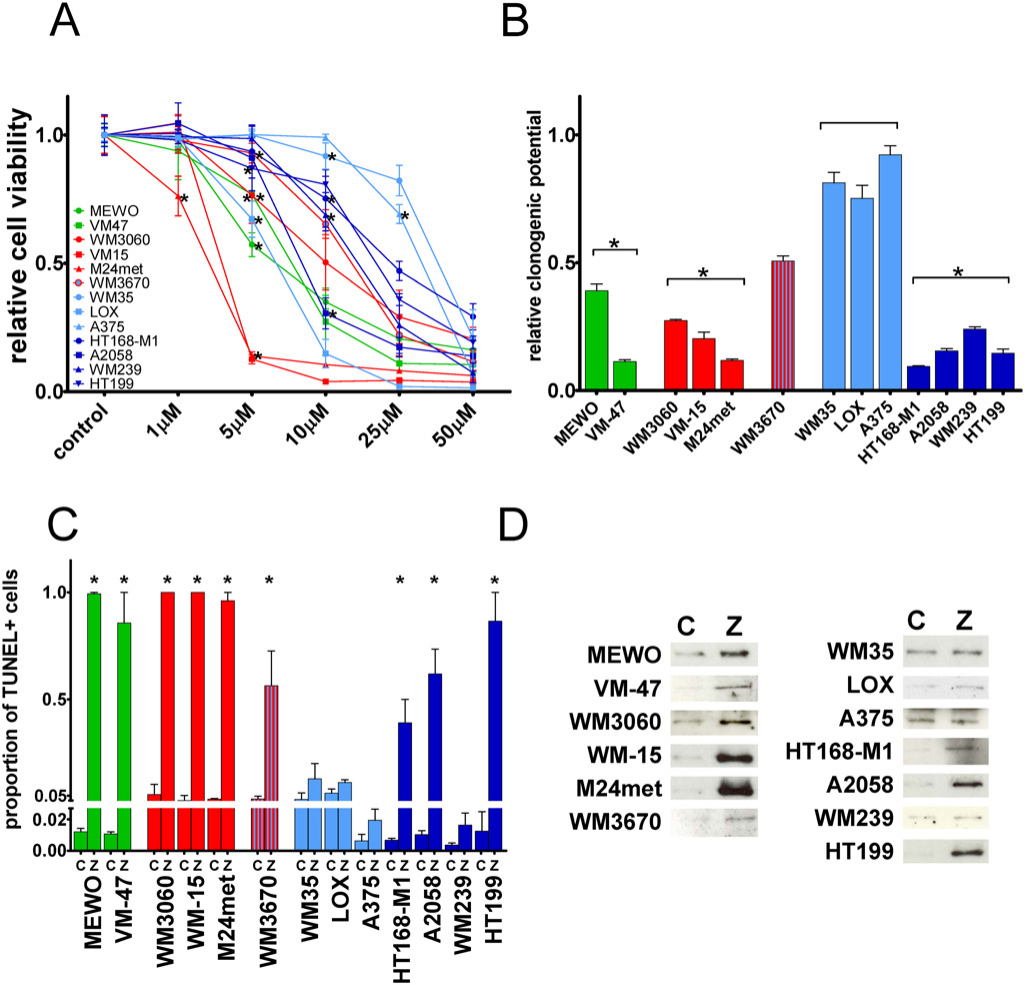
Cell viability, clonogenic growth and apoptosis in melanoma cells after zoledronic acid treatment. (**A**) Dose-response analysis of cell viability of human melanoma cell lines with different mutations after 72hs treatment with ZA measured by SRB assay; Most pronounced reduction in cell viability was observed in two NRAS mutant lines (**B**) Long-term effect of 10 days of 5 μM ZA treatment on clonogenic growth. Resistance was found in BRAF mutant and PTEN wild-type cells. Of note, NRAS and BRAF double mutant cells demonstrated intermediate sensitivity (**C**) Apoptosis induction after 25μM ZA treatment demonstrated by the proportion of TUNEL positive cells and (**D**) apoptosis induction of 72hs ZA treatment evaluated by the immunoblot of cleaved PARP. Limited apoptosis induction was found in BRAF mutant and PTEN wild-type cells and in the MDM2 over expressing WM239 cells. Colors green, red, blue, and dark-blue indicate triple wild-type, NRAS, BRAF, and BRAF mutant/PTEN-null mutational status of the cells, respectively. Data shown as average ± SEM are from at least 5 repeats. Asterisks indicate the lowest concentration of ZA treatment resulting in a significant difference with p < 0.05 from control by ANOVA and Dunnett’s post hoc test in the cell viability assay. Data shown as average ± SEM are from at least 3 measurements. Asterisks indicate significant difference of p < 0.05 between given mutational group and the BRAF mutant PTEN wild-type group by Kruskal-Wallis and Dunn’s post hoc test in the clonogenic assay, and p < 0.05 difference from the respective control by unpaired two tailed T test in the apoptosis assay. (C = control; Z = zoledronic acid).

Altogether thirteen human melanoma cell lines with different NRAS, BRAF and PTEN mutational status **(** Table 1 and S1 Fig.) were treated with the prenylation inhibitor zoledronic acid (ZA). Short-term effect of the treatment with different concentrations of ZA on cell proliferation of melanoma cells was measured by SRB-assay (Fig. 1A). ZA treatment clearly decreased cell viability in two NRAS mutant melanoma lines at relatively smaller doses. At the same time the lowest sensitivity was demonstrated by two BRAF mutant and PTEN wild-type lines. However, there was no equivocal correlation between oncogenic mutations and sensitivity to short-term ZA treatment on cell viability. Long-term effect of ZA treatment was measured by clonogenic assay (Fig. 1B). As expected NRAS mutant cells showed high sensitivity to ZA. Of note, sensitivity of BRAF mutant cells to ZA treatment depended on their PTEN mutational status. All four BRAF mutant/PTEN-null mutant cells were sensitive to long-term ZA treatment in contrast to three BRAF mutant and PTEN wild-type line that were essentially resistant against prenylation inhibition. The NRAS and BRAF mutant but PTEN wild-type WM3670 cells showed intermediate sensitivity. Importantly, when the reduction in clonogenic potential was averaged for the mutational subtypes, NRAS, BRAF mutant/PTEN-null and triple wild-type cells were significantly higher as compared to BRAF mutant PTEN wild-type cells (p < 0.0001 by Kruskal-Wallis and Dunn’s post hoc test).

In order to characterize the pro-apoptotic effect of ZA, TUNEL staining was performed after 72 hours treatment with 25μM ZA and the percentage of TUNEL positive cells evaluated (Fig. 1C). In general, there was a more profound pro-apoptotic effect of ZA treatment in NRAS and BRAF mutant/PTEN-null cells as compared to BRAF mutant PTEN wild-type cells. Of note, despite its limited clonogenic growth, the MDM2 overexpressing WM239 line failed to show robust apoptotic response. Increase in TUNEL positivity was seen also in both of the triple wild-type cell lines. The amount of apoptotic cells found in the TUNEL assay was in line with the reduction in cell viability measured in the clonogenic assay. Level of apoptosis induction after ZA treatment was also evaluated by the immunoblot staining of cleaved PARP, a target of the apoptosis effector caspase-3 (Fig. 1D). Similarly to the results of TUNEL assay, induced apoptosis correlated with the results of the long-term clonogenic growth measurements. The smallest degree of apoptosis induction could be shown in BRAF mutant and PTEN wild-type cells, and again, in WM239 cells.

### Migration is not inhibited by zoledronic acid in melanoma cells

**Fig 2:**
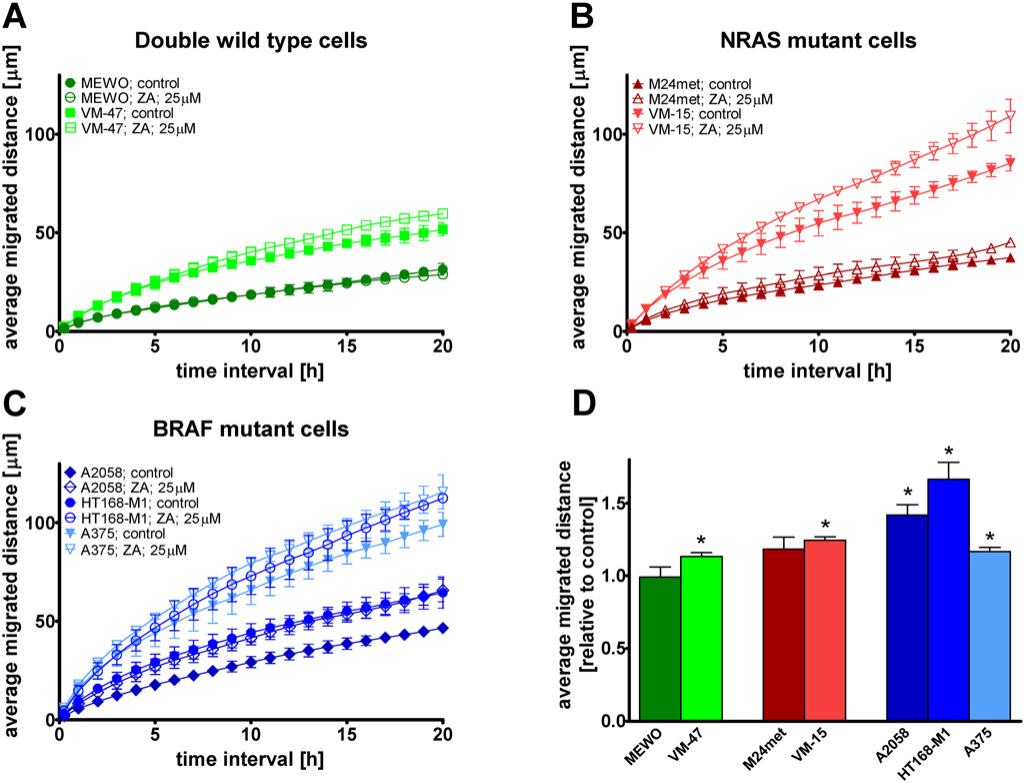
Cell migration after zoledronic acid treatment in melanoma cells. (**A-C**) Migrated distance as a function of time and (**D**) average migrated distance after zoledronic acid (ZA) treatment in melanoma cells measured by videomicroscopy. A profound and significant increase in migrated distance was found in all of the BRAF mutant cells. A modest but significant increase in migration was found in VM-47 triple wild-type and VM-15 NRAS mutant cells. Colors blue, red and green indicate BRAF, NRAS mutation and wild-type for these genes, respectively. Data shown as average ± SEM are from at least three independent measurements. Asterisks indicate significant difference of p < 0.05 from the respective control with unpaired two-tailed T test.

Videomicroscopy measurements were used to evaluate the effect of ZA treatment on migration in one BRAF mutant and PTEN wild-type, two BRAF mutant and PTEN-null, two NRAS mutant and two triple wild-type melanoma cell lines. Average migrated distance of the cells—grouped by mutational status—was depicted as a function of time interval from 15 minutes to 20 hours (Fig. 2 A–C). Furthermore, relative average migrated distance for 24 hours was calculated for each cell line (Fig. 2 D). ZA treatment increased the migratory activity of BRAF-mutant cells to a much higher extent compared to NRAS mutant and double wild-type cells. Significant increase in average migrated distance was measured in VM-47, VM-15, A375, A2058 and HT168-M1 cells by unpaired t-test (p = 0.0086, p = 0.0116, p = 0.0091, p = 0.0495, and p = 0.0065, respectively). When migration was compared by mutational status, the increase in average migrated distance was significant (p = 0.0427 by unpaired t-test) in BRAF mutant cells.

### Effect of ZA on Erk and S6 activation in melanoma cells

**Fig 3:**
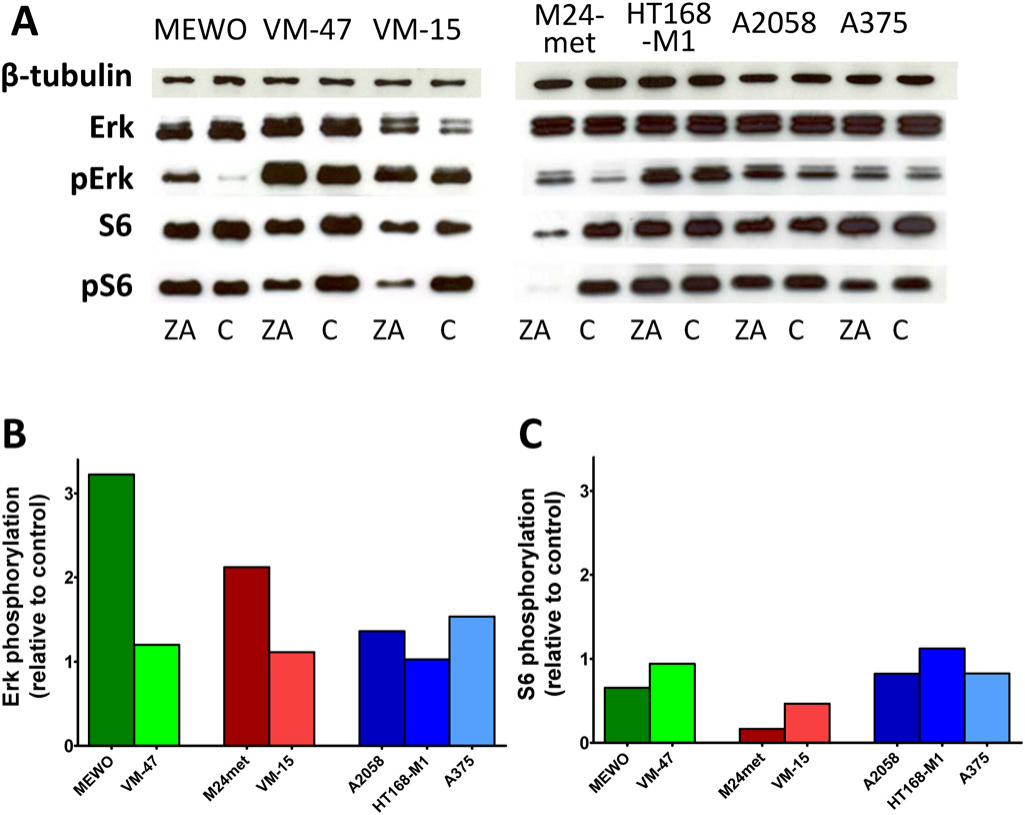
Activation of downstream elements of the RAS/RAF pathway in melanoma cells after zoledronic acid treatment. (**A**) Representative blots of the effect of 48hs zoledronic acid (ZA) treatment on the activation of Erk1/2 and S6. (**B**) Quantification of the effect of ZA treatment on the activation of Erk1/2. Treatment with ZA resulted in robust increase in the phosphorylation of Erk1/2 in MEWO and M24met cells. (**C**) Quantification of the effect of ZA treatment on the activation of S6. After the treatment with ZA, decreased activation of S6 proteins was found only in NRAS mutant M24met and VM-15 cells. Colors blue, red and green indicate BRAF, NRAS mutation and wild-type for these genes, respectively. (C = control; ZA = zoledronic acid).

To investigate the effect of ZA on the major signaling cascades that are regulated by oncogenic BRAF or NRAS we performed immunoblot analysis of the activation of two major downstream effectors of the RAS-RAF-MEK and the PI3K-AKT-mTOR pathway, the Erk1/2 and the ribosomal protein S6 proteins, respectively (Fig. 3). Surprisingly, 48 hours of ZA treatment increased activation of Erk1/2 in MEWO and M24met cells and to a smaller extent in BRAF mutant cells. The treatment with ZA resulted in decreased activation of S6 in NRAS mutant cells. The more sensitive M24met cells showed higher decrease than the less sensitive NRAS mutant VM-15 cells. Furthermore, both NRAS mutant cell line had lower level of Akt phosphorylation after ZA treatment (S2 Fig.).

### Effect of combination of zoledronic acid (ZA) and cytotoxic compounds *in vitro*

To investigate the potential synergistic effect of prenylation inhibition with cytotoxic therapy, ZA treatment was applied concurrently with DTIC or cisplatin on three human melanoma cell lines with BRAF or NRAS mutation or harboring no mutations of these genes.

**Fig 4:**
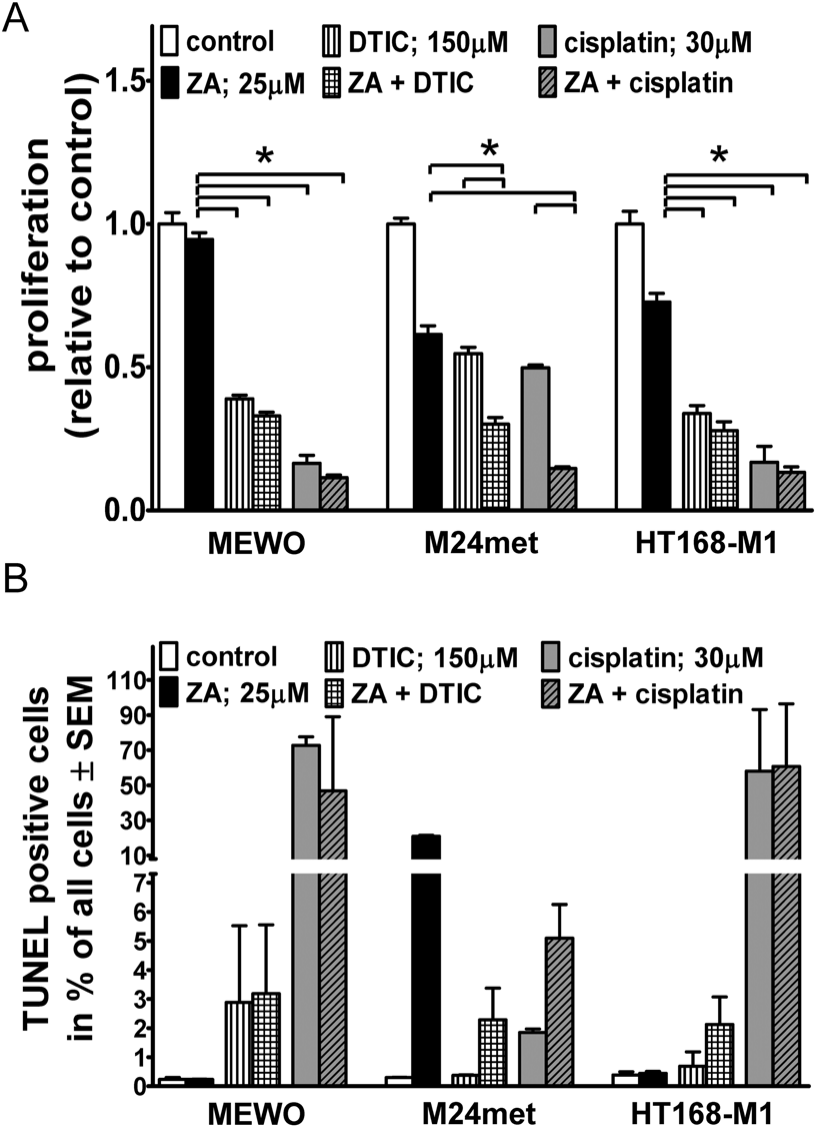
Proliferation and apoptosis in melanoma cells after combination treatments. (**A**) Both cytotoxic compounds reduced proliferation significantly after 48hs of treatment in three cell lines irrespective of oncogenic mutation using SRB assay. An additive effect of ZA and cytotoxic treatment was measured in the NRAS mutant M24met cells. (**B**) Proportion of TUNEL positive cells after combined treatment with ZA and standard cytotoxic therapy (DTIC or cisplatin). No synergism was observed in any of the cell lines investigated. Data shown as average ± SEM are results of four independent measurements. Asterisks indicate significance of p < 0.05 with Kruskal-Wallis and Dunn’s multiple comparison tests.

DTIC and cisplatin significantly reduced proliferation in all examined cell lines (p < 0.05 by Kruskal-Wallis and Dunn’s post hoc test) based on the SRB assay (Fig. 4A). There was a significantly increased reduction in proliferation measured after combination treatment as compared to the single treatment in the NRAS mutant M24met line. In order to further evaluate the potential interaction we performed further combination experiments with an additional NRAS mutant line (VM15). We could only demonstrate additive effects with either of the compounds (S3 Fig.).

In order to characterize the proapoptotic effect of these drugs, we performed TUNEL assay (Fig. 4B). High proapoptotic effect of single agent ZA treatment in NRAS mutant cells was found, and no additive effect with either cytotoxic compound was seen. DTIC had no effect on ZA induced increase in cell migration in BRAF mutant cells (S4 Fig.). In NRAS mutant and double wild-type cells neither the single nor the combined treatment changed migratory activity.

**Fig 5:**
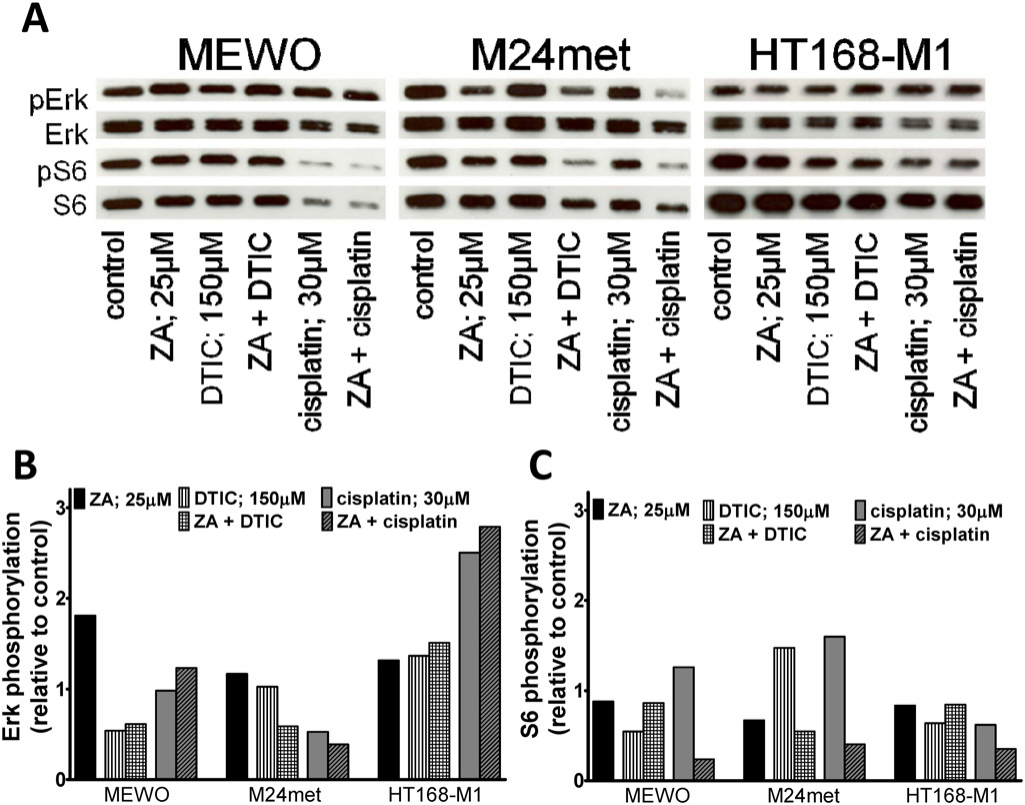
Activation of downstream elements of the RAS/RAF pathway in melanoma cells after combination treatments. (**A**) Representative blots of the effect of 12hs zoledronic acid (ZA) and/or DTIC/cisplatin treatment on the activation of Erk1/2 and S6. (**B**) Quantification of the effect of ZA and/or DTIC/cisplatin treatment on the activation of Erk1/2. Treatment with cytotoxic agents decreased the activation of Erk1/2 in double wild type MEWO and NRAS mutant M24met cells. DTIC or cisplatin and combined treatment resulted in the increase of Erk1/2 activation in BRAF mutant HT168-M1 cells. (**C**) Quantification of the effect of ZA and or DTIC/cisplatin treatment on the activation of S6. DTIC or cisplatin treatment increased S6 activation in HT168-M1 cells. S6 activation was also increased in M24met and in the combination treatment with cisplatin also in MEWO cells. Synergism between ZA and cytotoxic treatment was seen only in M24met cells if treated with DTIC and ZA and MEWO cells after the treatment with cisplatin and ZA.

To investigate the effect of ZA in combination with cytotoxic drugs (DTIC or cisplatin) on the major signaling cascades downstream of BRAF or NRAS immunoblot analysis of the activation the proteins Erk1/2 and the ribosomal protein S6 was performed (Fig. 5). 12 hours treatment with cytotoxic agents decreased the activation of Erk1/2 in MEWO and M24met cells, double wild-type and NRAS mutant, respectively. In contrast DTIC or cisplatin and combined treatment resulted in the increase of Erk1/2 and S6 activation in the BRAF mutant HT168-M1 cells. S6 activation was increased in M24met and upon treatment with cisplatin also in MEWO cells. Importantly synergism between ZA and cytotoxic treatment was seen only in M24met cells if treated with DTIC and ZA and MEWO cells after the treatment with cisplatin and ZA.

### Effect of zoledronic acid (ZA) treatment *in vivo*

**Fig 6:**
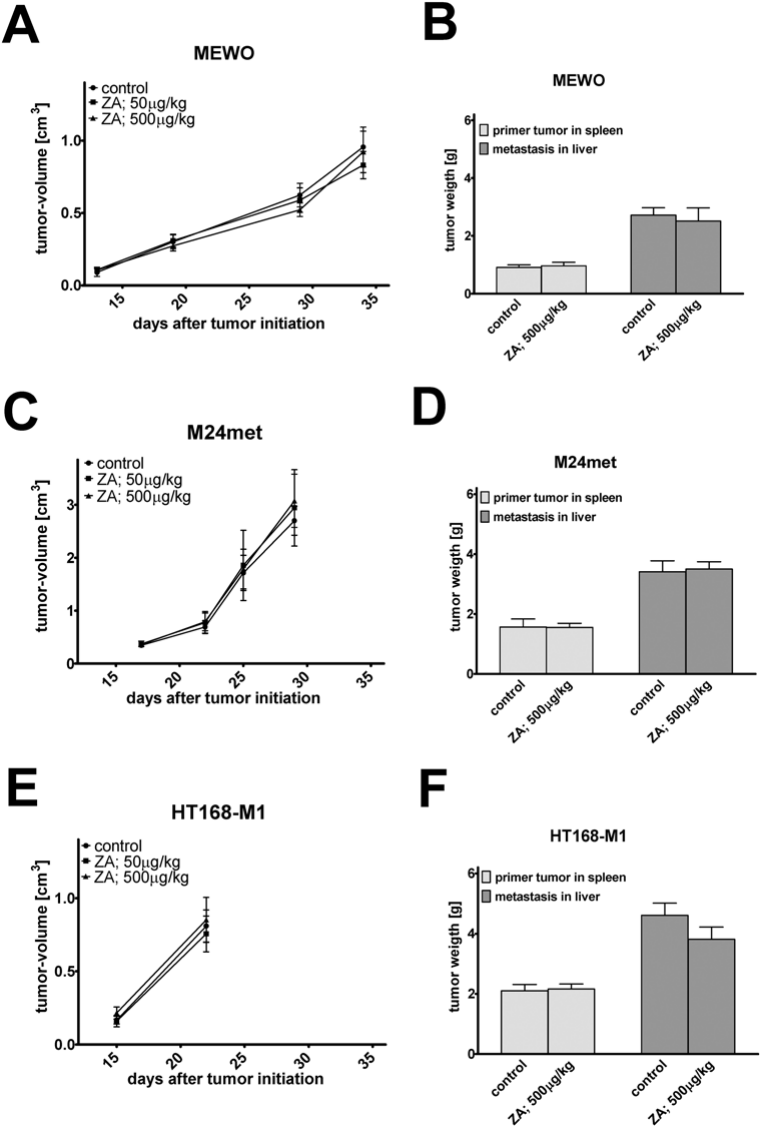
*In vivo* effects of zoledronic acid treatment. Effect of zoledronic acid (ZA) treatment using *in vivo* subcutaneous xenograft model of human melanoma cells in SCID mice (**A, C, E**). ZA treatment failed to show effects in the subcutaneous growth of melanoma cells with either mutation. (**B, D, F**) Effect of ZA treatment using *in vivo* spleen liver colonization model of human melanoma cells in SCID mice. ZA did not inhibit the primary tumor or metastatic growth of melanoma cells. Data shown as average ± SEM.

To assess the effect of zoledronic acid on primary tumor growth we injected subcutaneously human melanoma cells into the flank of SCID mice (Fig. 6A, C and E). ZA treatment failed to reduce the growth of melanoma cells. Of note, BRAF mutant/PTEN-null HT168-M1 cells showed a very rapid growth rate after the initial lag phase that prompted the early termination of the experiment. To investigate the metastasis related effects of ZA, we used the spleen-to-liver colonization model in SCID mice (Fig. 6B, D and F). ZA could not inhibit either the primary or the metastatic growth of melanoma cells.

## Discussion

In earlier investigations, despite great promises, farnesyltransferase inhibitors (FTIs) showed limited efficacy in monotherapy clinical trials [2, 25, 26]. A number of studies have investigated why targeting the major posttranslational molecular mechanism is not effective [58–60]. One important mechanism of FTI-resistance is the alternative geranylgeranylation of K-Ras and possibly N-Ras (but not H-Ras) [27–29]. Due to this alternative mechanism in order to efficiently prevent RAS activation the dual inhibition of farnesyltransferase and geranylgeranylase is necessary [30]. According to our current knowledge, the prenylation of mutant N-Ras is also required to exert its oncogenic function [21]. Zoledronic acid (ZA), a nitrogen containing bisphosphonate inhibits the intracellular key enzyme of the mevalonate pathway, namely the farnesyl diphosphate syntase and thus interfering with both farnesylation and geranylgeranylation. Accordingly we investigated the response of melanoma cells to ZA. First we examined ZA’s short-term (72 h) effect on melanoma cells with various NRAS, BRAF and PTEN mutation status, but the role of these mutations on treatment response was not equivocal. Especially at lower concentrations a considerable proportion of cells remained vital. However, long-term (10 days) treatment with low concentration (5 μM) ZA had strong antiproliferative effect on triple wild-type, NRAS mutant and BRAF mutant/PTEN-null cell lines. We found also apoptosis induction in triple wild-type, NRAS mutant and BRAF mutant/PTEN-null cell lines with the exception of WM239 cells. WM239 cells harbor no p53 mutation, but express extremely high amount of mdm2, that is responsible for the degradation of p53 and can therefore lead to an extremely low amount of apoptosis despite the decrease in cell viability [61]. In case of BRAF mutant PTEN wild-type cells, such as A375, we found only limited decrease in proliferation *in vitro* below the 25μM concentration similar to the study by Forsea et al.[49]. Interestingly, BRAF mutant/PTEN-null melanoma cells showed much higher sensitivity to ZA treatment as PTEN wild-type BRAF mutant cells. In line with our observation, the effect of PTEN loss on therapeutic sensitivity of melanoma cells has been demonstrated earlier in case of other drugs. Interestingly, PTEN loss conferred resistance against EGFR inhibitors in colorectal and lung cancer [62, 63], against herceptin in breast cancer [64] or even BRAF inhibitors in melanoma [65].

In contrast to the *in vitro* proliferation inhibiting effect of ZA, we found no inhibition of primary tumor growth in the subcutaneous xenograft models of investigated human melanoma cells *in vivo*. The lack of antitumor effect of ZA *in vivo* on the NRAS mutant and BRAF mutant and PTEN null melanoma cells may be explained by the fact, that around 50–60% of ZA accumulates in the skeleton and the remaining part is excreted in the first 24hs after drug administration [66, 67].

*In vitro* treatment with ZA resulted in increased migration activity of certain melanoma cells that may confer increased invasiveness or metastatic potential. Thus, we addressed this question in the *in vivo* spleen-to-liver colonization model that reflects certain aspect of metastasis formation. However, we found no change in the liver colonization capacity after zoledronic acid treatment.

In order to assess the role of the major oncogenic signaling pathways we measured the activation of the ribosomal protein S6 and the proteins Erk1/2 by immunoblot analysis In the two NRAS mutant cell line with robust short-term response to ZA, decreased S6 activation was found. However, we found no correlation between S6 activation levels and apoptosis induction or long-term clonogenic potential changes. Interestingly, the activation of Erk1/2 showed an inverse correlation with cell motility similarly to former observations on primary endothelial cells where the deletion of Erk1/2 resulted in an inhibition of proliferation and migration [68]. A potential mechanism by which Erk1/2 activation can induce cell migration has been described by Carragher and colleagues [69]. They demonstrated that FAK recruits activated Erk to focal adhesion to activate calpain2. Subsequently, this protease contributes to the disassembly of focal adhesion complexes and thus stimulating cell migration.

Furthermore in our study we have explored the feasibility of combining standard cytotoxic treatments (DTIC or cisplatin) with ZA. Importantly, we found that the efficacy of combination treatment was also mutation dependent. An additive effect in inhibition of cell viability was only found in NRAS mutant but not in BRAF mutant or double wild-type cells. Interestingly, this additive effect of combination treatment was not seen in the apoptosis of NRAS mutant cells. In an earlier study, pamidronate, a nitrogen-containing bisphosphonate similar to ZA, failed to show synergetic effect in a combination regime with DTIC in melanoma cells *in vitro* [48]. However, the study did not include melanoma cells with known NRAS mutation. More recently, Niessner et al [70] demonstrated significant synergetic effect of lonafarnib and sorafenib (a nonselective kinase inhibitor) in melanoma cells with NRAS and BRAF mutations or double wild-type cells. Our observation is also in line with studies from other tumor types where ZA has already been found to be additive or synergistic with certain chemotherapeutic drugs [71–73].

In summary our findings suggest that prenylation inhibition may be able to target melanoma cells with mutant NRAS or with mutant BRAF and PTEN. Importantly, benefit of prenylation inhibition may strongly depend on the driver oncogenic mutations present in a tumor. Furthermore, based on our preclinical findings, additional prenylation inhibitors need to be developed or investigated that have improved pharmacological properties in order to be effective against NRAS mutant malignant melanoma.

## Supporting Information

S1 Fig Immunoblot staining for PTEN expression of all 13 cell lines. Representative blots for the evaluation of PTEN expression in the examined melanoma cell lines.
(TIF)

S2 Fig Immunoblot staining for p-Akt for two NRAS mutant cell lines. Representative blots of the effect of 48hs zoledronic acid (ZA).
(TIF)

S3 Fig Cell viability in NRAS mutant melanoma cells (M2met and VM-15) after combination treatments. Effects of combined treatment with ZA and DTIC (**A, B**) or cisplatin (**C, D**) were investigated using 48hs treatment and SRB assay. Although some additive effect, no clear synergism could be observed.
(TIF)

S4 Fig Migration in melanoma cells after combined treatment with zoledronic acid (ZA) and DTIC. ZA treatment increased the migratory activity of BRAF mutant cells, but interestingly, DTIC had no effect on ZA induced changes in cell migration. In NRAS mutant and double wild-type cells neither the single nor the combined treatment changed migration activity. Data shown as average ± SD are results of three independent measurements. Asterisks indicate significance of p < 0.05 by Kruskal-Wallis and Dunn’s multiple comparison test.
(TIF)
