# Supplementary figures and images for: Prenylation Inhibition-Induced Cell Death in Melanoma: Reduced Sensitivity in BRAF Mutant/PTEN Wild-Type Melanoma Cells
Source: PLoS One. 2015 Feb 3;10(2):e0117021. doi: 10.1371/journal.pone.0117021 (PMC4315579; doi:10.1371/journal.pone.0117021)

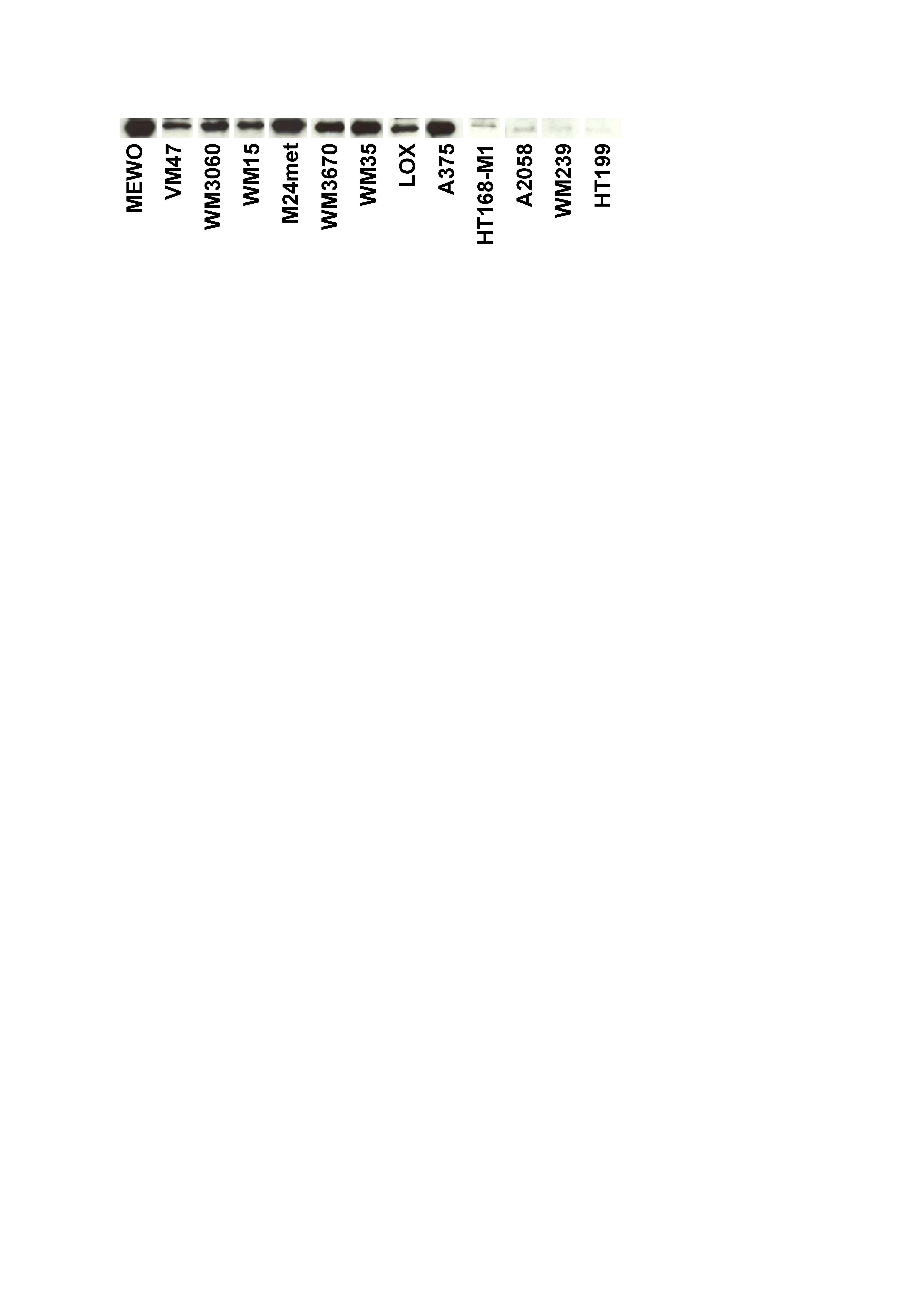

Supplement: S1 Fig — Representative blots for the evaluation of PTEN expression in the examined melanoma cell lines. (TIF) [file pone.0117021.s001.tif]

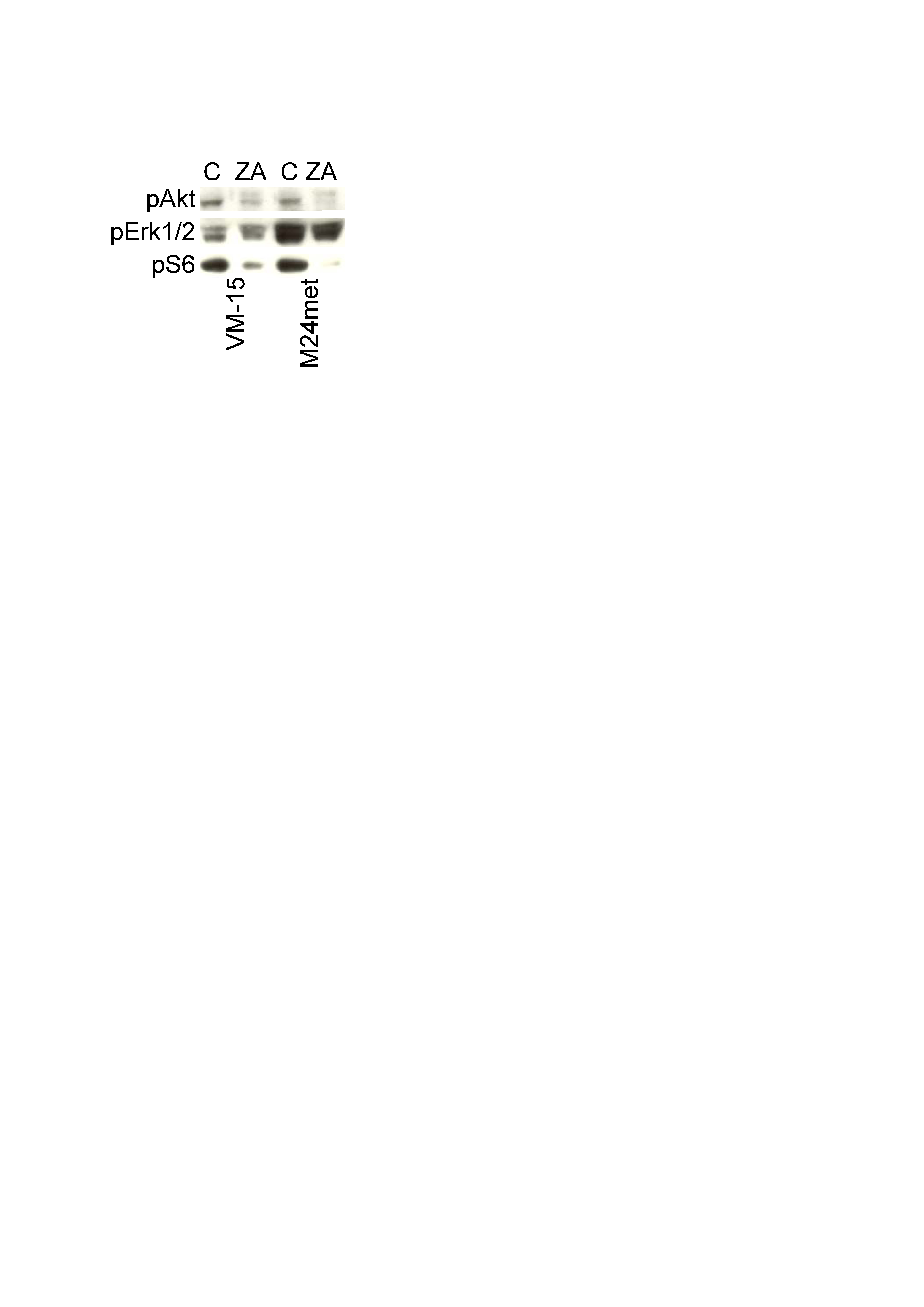

Supplement: S2 Fig — Representative blots of the effect of 48hs zoledronic acid (ZA). (TIF) [file pone.0117021.s002.tif]

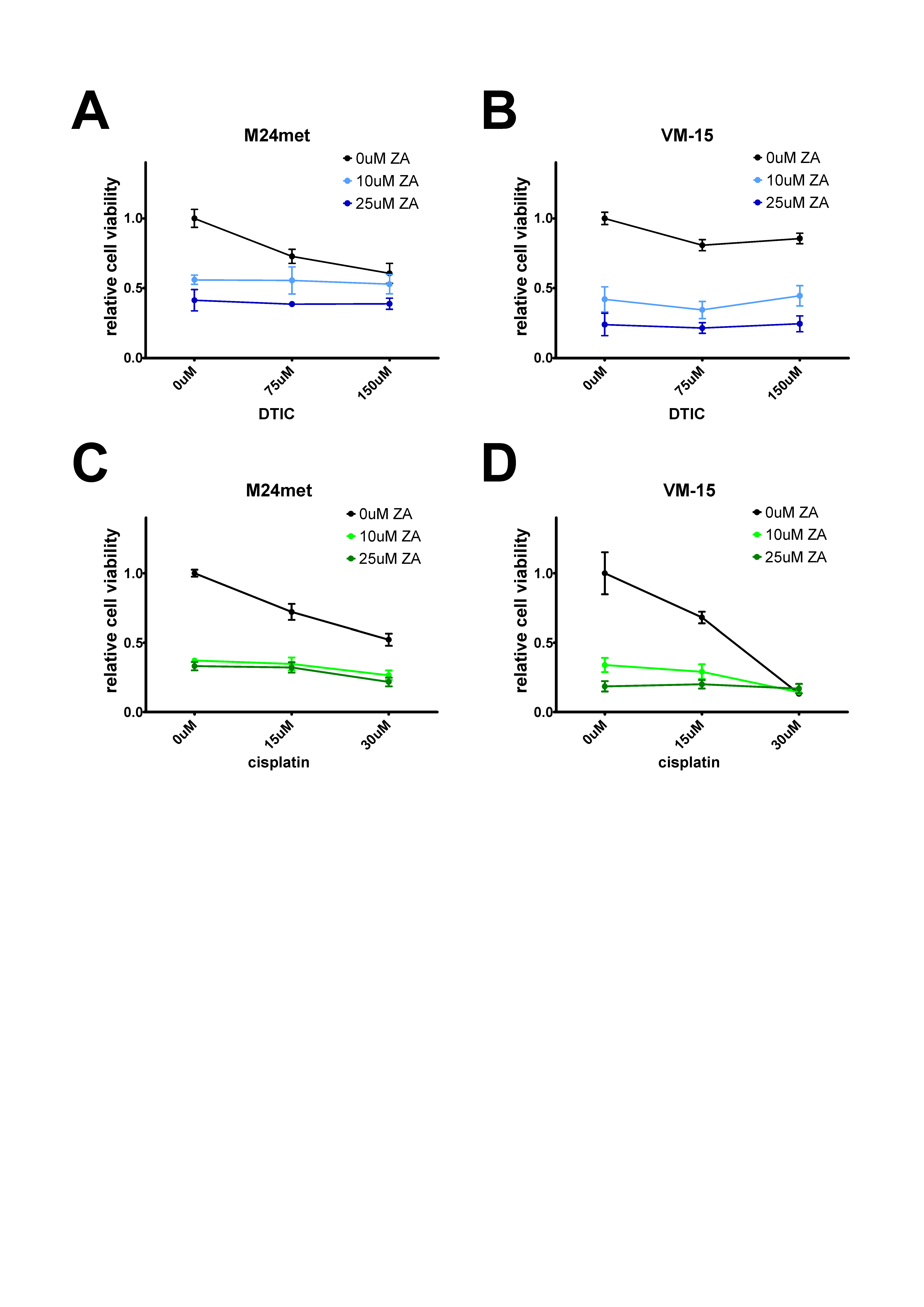

Supplement: S3 Fig — Effects of combined treatment with ZA and DTIC (A, B) or cisplatin (C, D) were investigated using 48hs treatment and SRB assay. Although some additive effect, no clear synergism could be observed. (TIF) [file pone.0117021.s003.tif]

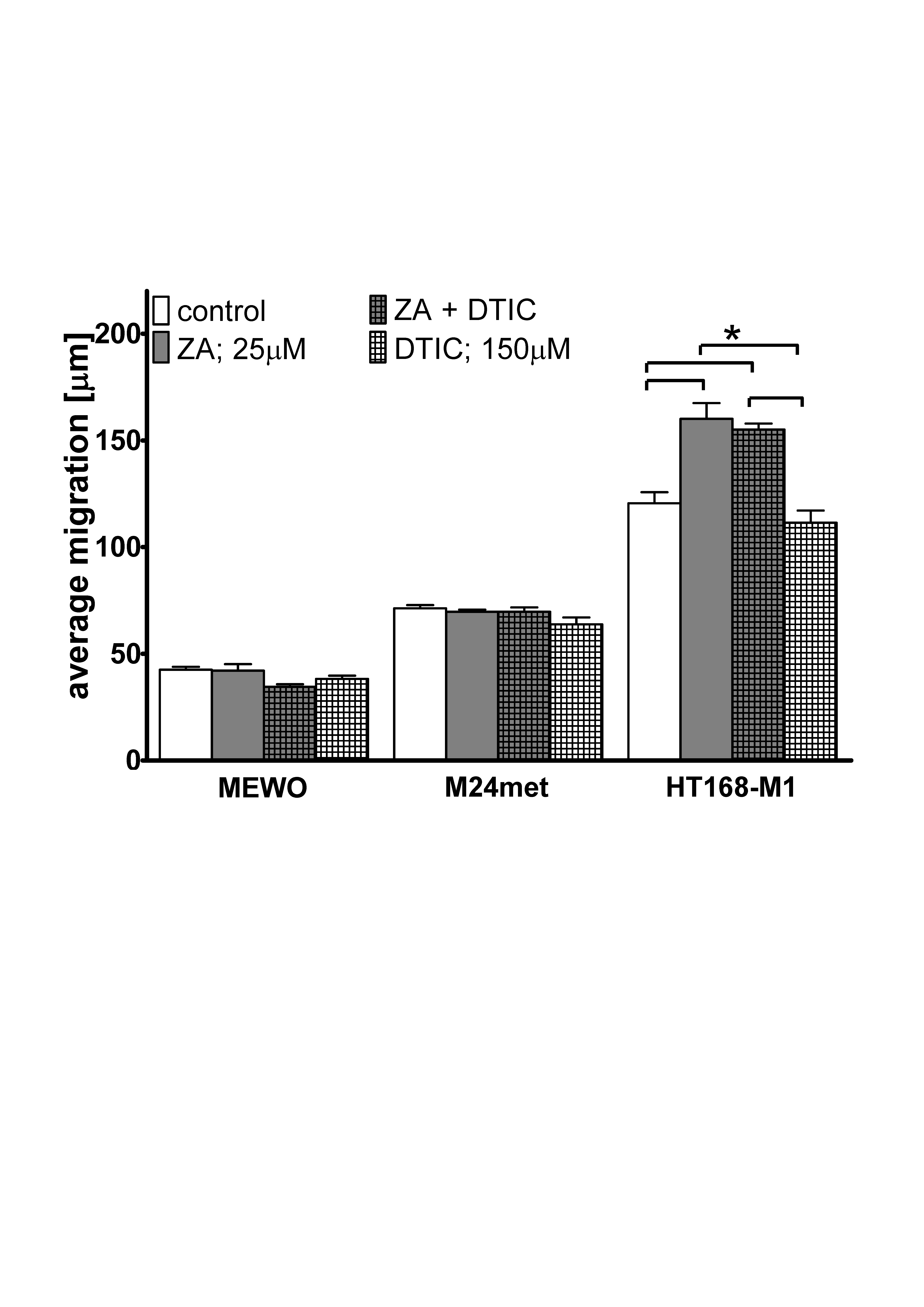

Supplement: S4 Fig — ZA treatment increased the migratory activity of BRAF mutant cells, but interestingly, DTIC had no effect on ZA induced changes in cell migration. In NRAS mutant and double wild-type cells neither the single nor the combined treatment changed migration activity. Data shown as average ± SD are results of three independent measurements. Asterisks indicate significance of p < 0.05 by Kruskal-Wallis and Dunn’s multiple comparison test. (TIF) [file pone.0117021.s004.tif]
